# An Assessment of the Molecular Diversity of Ticks and Tick-Borne Microorganisms of Small Ruminants in Pakistan

**DOI:** 10.3390/microorganisms8091428

**Published:** 2020-09-17

**Authors:** Abdul Ghafar, Adil Khan, Alejandro Cabezas-Cruz, Charles G. Gauci, Sadaf Niaz, Sultan Ayaz, Lourdes Mateos-Hernández, Clemence Galon, Nasreen Nasreen, Sara Moutailler, Robin B. Gasser, Abdul Jabbar

**Affiliations:** 1 Department of Veterinary Biosciences, Melbourne Veterinary School, Faculty of Veterinary and Agricultural Sciences, The University of Melbourne, Werribee 3030, Victoria, Australia; aghafar@student.unimelb.edu.au (A.G.); zoologyawkum@gmail.com (A.K.); charlesg@unimelb.edu.au (C.G.G.); robinbg@unimelb.edu.au (R.B.G.); 2 Department of Zoology, Faculty of Chemical and Life Sciences, The Abdul Wali Khan University, Mardan 23200, Khyber Pakhtunkhwa, Pakistan; sadaf@awkum.edu.pk (S.N.); sultanayaz64@awkum.edu.pk (S.A.); nasreen@awkum.edu.pk (N.N.); 3 UMR BIPAR, INRAE, ANSES, Ecole Nationale Vétérinaire d’Alfort, Université Paris-Est, 94700 Maisons-Alfort, France; alejandro.cabezas@vet-alfort.fr (A.C.-C.); lourdes.mateoshernandez@anses.fr (L.M.-H.); clemence.galon@anses.fr (C.G.); sara.moutailler@anses.fr (S.M.)

**Keywords:** tick, *Anaplasma*, goat, *Haemaphysalis*, microfluidic real-time PCR, *Rhipicephalus*, *Rickettsia*, sheep, *Theileria*, Pakistan

## Abstract

This study investigated ticks and tick-borne microorganisms of small ruminants from five districts of the Federally Administered Tribal Area (FATA) of Pakistan. Morphological (*n* = 104) and molecular (*n* = 54) characterization of the ticks revealed the presence of six ixodid ticks: *Rhipicephalus* (*Rh*.) *haemaphysaloides*, *Rh. microplus*, *Rh. turanicus*, *Haemaphysalis* (*Hs*.) *punctata*, *Hs. sulcata* and *Hyalomma anatolicum*. Phylogenetic analyses of nucleotide sequence data for two mitochondrial (16S and cytochrome *c* oxidase 1) and one nuclear (second internal transcribed spacer) DNA regions provided strong support for the grouping of the six tick species identified in this study. Microfluidic real-time PCR, employing multiple pre-validated nuclear and mitochondrial genetic markers, detected 11 potential pathogens and endosymbionts in 72.2% of the ticks (*n* = 54) tested. *Rickettsia* (*R.*) *massiliae* was the most common pathogen found (42.6% of ticks) followed by *Theileria* spp. (33.3%), *Anaplasma* (*A.*) *ovis* and *R. slovaca* (25.9% each). *Anaplasma centrale*, *A. marginale*, *Ehrlichia* spp., *R. aeschlimannii*, *R. conorii* and endosymbionts (*Francisella*- and *Coxiella*-like) were detected at much lower rates (1.9–22.2%) in ticks. Ticks from goats (83.9%) carried significantly higher microorganisms than those from sheep (56.5%). This study demonstrates that ticks of small ruminants from the FATA are carrying multiple microorganisms of veterinary and medical health significance and provides the basis for future investigations of ticks and tick-borne diseases of animals and humans in this and neighboring regions.

## 1. Introduction

Ticks are obligate blood-feeding ectoparasites of animals and humans that are distributed globally [1]. They can affect their hosts either directly by causing tick-associated stress, irritation, allergy, anemia, weight loss and paralysis or indirectly by transmitting numerous pathogenic microorganisms including bacteria, fungi, protozoa, rickettsiae, spirochetes and/or viruses [1,2,3]. In production animals such as cattle, buffaloes, goats and sheep, tick-borne protozoal (babesiosis and theileriosis) and rickettsial (anaplasmosis and cowdriosis) diseases cause major health problems as well as production and economic losses mainly in subtropical and tropical regions [1].

Pakistan is a subtropical country where the majority of the rural population is dependent upon livestock including small ruminants for their food and livelihood (Pakistani goat and sheep population: 31.2 million *Ovis aries*; 78.2 m *Capra hircus*), particularly in the Federally Administered Tribal Area (FATA) of the north-western part of the country [4,5]. The FATA is located near the Pak-Afghan border and represents one of the least-developed regions in Pakistan due to political unrest and prolonged military crises over the last 50 years. These areas consist of seven tribal agencies (districts) and six frontier regions, which have been recently merged [6]. Nomadic pastoralism is a common practice in this region and > 70% of the human population derives their livelihood from livestock farming [7]. Due to the poor infrastructure, limited resources and inadequate access to veterinary services, tick-borne diseases (TBDs) of humans and animals have a major impact in this region [7].

Although a number of studies have assessed the prevalence of ticks and tick-borne diseases (TTBDs) of small ruminants in different areas of Pakistan [8,9,10,11,12,13,14,15,16,17,18,19], there is a paucity of information from the FATA. Moreover, no study has yet investigated the presence, prevalence and diversity of tick-borne pathogens (TBPs) of small ruminants in this region. Recently, Khan et al. [20] assessed tick burdens on small ruminants in the FATA using morphological methods and found three main genera of ixodid ticks (*Haemaphysalis*, *Hyalomma* and *Rhipicephalus*) but these ticks and associated TBPs were not further characterized in detail using molecular tools.

Specific identification is pivotal for understanding the epidemiology of, and developing effective control strategies for, TTBDs [21]. However, morphological methods do not allow the identification of immature, engorged or damaged tick specimens [22,23]. By contrast, molecular methods provide an alternative and complementary approach for the identification of ticks [24], which employ the characterization of genetic markers such as the mitochondrial cytochrome *c* oxidase subunit I (*cox*1) and 16S ribosomal RNA genes [25,26]. As ticks usually harbor and transmit commensals and numerous pathogens, some of which can be of public health significance (e.g., *Coxiella burnetii* and Crimean-Congo hemorrhagic fever virus) [27,28,29], it is important to detect these microorganisms in ticks to ascertain their prevalence. However, conventional diagnostic methods such as microscopic examination of thin and thick smears usually detect few target pathogens or microorganisms and have a lower sensitivity and specificity than molecular approaches [30]. Therefore, testing ticks as well as their animal hosts using polymerase chain reaction (PCR) based methods for the detection of TBPs and/or commensals provides distinct advantages over conventional detection methods. Recently, a micro-chip-based microfluidic real-time PCR technique was developed to detect and differentiate up to 96 microorganisms per tick in a single PCR procedure [31]. This method has been proven to be well-suited for rapid and large-scale epidemiological and surveillance studies [28,31,32,33,34,35,36] and is anticipated to be an invaluable tool for the detection of microorganisms in ticks from regions such as the FATA.

In the present study, we employed both conventional and PCR-based tools to investigate the diversity of tick taxa from small ruminants and their associated TBPs and/or commensals in the FATA of Pakistan.

## 2. Materials and Methods 

### 2.1. Study Area and Tick Samples

**Figure 1:**
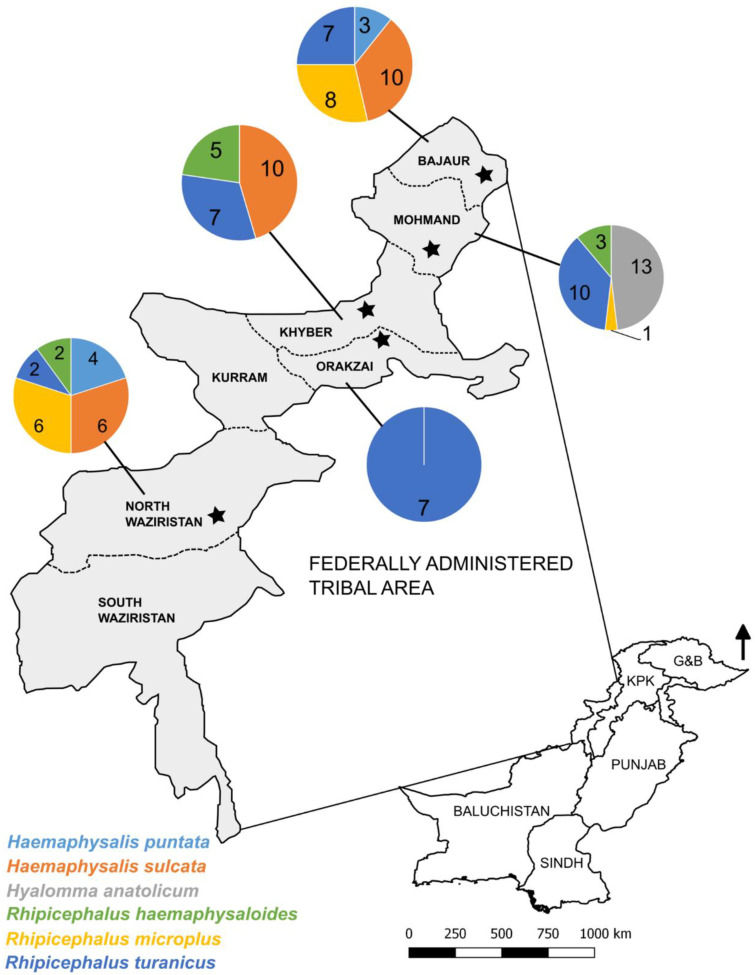
Map of Pakistan (bottom right) and the Federally Administered Tribal Area (FATA) (grey-colored areas on top left) showing the tribal districts (starred) included in this study. Different colors in each pie chart indicate the distribution of tick species identified from each district.

The FATA represents seven tribal districts in the north-western part of Pakistan (Figure 1). This region has a monsoonal climatic zone in the East and a Mediterranean one in the west such that seasons and rainfall can vary markedly across regions. In agriculture, livestock production is one of the main sources of subsistence for two-thirds of the population. The estimated population of small ruminants in the FATA is ~5.5 million [37].

A convenience sampling method was used to collect 104 hard ticks from sheep (*n* = 23) and goats (*n* = 31) from the five tribal districts of Bajaur, Khyber, Mohmand, North Waziristan and Orakzai (Figure 1; Table 1). Ticks were collected using tweezers from various body parts of the animals including ears, neck and the perineal region. Following tick collection, the specimens from individual animals were fixed in 70% ethanol in separate Eppendorf tubes, labelled and stored at an ambient temperature. The collection of ticks from the animals was approved by the Animal Ethics Committee of the Abdul Wali Khan University.

### 2.2. Morphological Identification of Ticks and DNA Extraction

Using a dissecting microscope (Olympus SZ40, Olympus Corporation, Tokyo, Japan), individual ticks were morphologically identified to species employing dichotomous keys [23,38,39]. Subsequently, DNA was extracted from 54 individual ticks representing each host species in each tribal district. Briefly, following rehydration, each tick was cut in half longitudinally. One half of each tick was washed three times in 15 mL of H_2_0 and then diced finely with a sterile scalpel blade. DNA was extracted using a DNeasy Blood and Tissue Kit (Qiagen, Hilden, Germany) following the manufacturer’s protocol, except that the proteinase K digestion step was for 24–48 h at 56 °C. The DNA concentration of each sample was estimated using a Nanodrop ND1000. 

### 2.3. Molecular Characterization of Ticks

Two mitochondrial loci (*cox*1 and 16S) were amplified separately from each individual DNA sample representing each tick specimen using previously published primers [25,40] (see Supplementary Materials Table S1) by conventional PCR in a thermal cycler (T100, BioRad). Additionally, a region of the second internal transcribed spacer (ITS-2) of nuclear ribosomal DNA was amplified using published primers to provide further differentiation of *Hyalomma* or *Rhipicephalus* species [41] (see Supplementary Materials Table S1). All PCRs were carried out in a volume of 25 µL containing 3.12 mM of each deoxynucleotide triphosphate (dNTP), 6.25 pmol of each primer and 10 mM Tris-HCl (pH 8.4), 50 mM KCl, MgCl_2_ at 75 mM (16S reactions), 100 mM (*cox*1 reactions) or 150 mM (ITS-2 reactions) and 0.6 U of Go*Taq* flexi DNA polymerase (Promega, Madison, WI, USA). Known positive (*Rh. sanguineus* DNA, Xng) and negative (milli-Q H_2_O) controls were included in each PCR run. Aliquots (5 µL) of individual amplicons were examined on 1.5% (*w/v*) agarose gels stained with GelRed (Biotium, Fremont, CA, USA) and then photographed using a GelDoc system (BioRad, Hercules, CA, USA). 

For each locus, amplicons were purified using a Favorgen Gel/PCR purification mini kit (Favorgen, Ping-Tung, Taiwan) and DNA concentration was measured using a spectrophotometer (ND-1000, NanoDrop, Wilmington, DE, USA). Aliquots (5 µL) of individual amplicons were subjected to automated and bidirectional (Sanger) sequencing using the same primers (individually) as employed in the primary PCR (Supplementary Materials Table S1).

### 2.4. Microfluidic PCR-Based Detection of Tick-Borne Microorganisms

Individual DNA samples from individual ticks (*n* = 54) were subjected to microfluidic amplification of microorganisms using a 48.48 dynamics array in a Bio-Mark™ real-time PCR system (Fluidigm, CA, USA) as described previously [31]. Target microorganisms (and Gene/DNA markers) were *Anaplasma* (*A*.) species (spp.) (16S), *A. marginale* (*msp*1), *A. platys* (*groEL*), *A. phagocytophilum* (*msp*2), *A. ovis* (*msp*4), *A. centrale* (*groEL*), *A. bovis* (*groEL*), Apicomplexa spp. (18S), *Babesia* (*B.*) *microti* (*CCTeta*), *B. canis* (18S), *B. ovis* (18S), *B. bovis* (*CCTeta*), *B. caballi* (*rap*1), *B. bigemina* (18S), *B. divergens* (*hsp*70), *B. vulpes* (*cox*1), *Bartonella* (*Ba.*) spp. (*ssrA*), *Ba. henselae* (*pap*31), *Borrelia* (*Bo*.) spp. (23S), *Bo. burgdorferi* s.s. (*rpoB*), *Bo. garinii* (*rpoB*), *Bo. afzelii* (*fla*), *Bo. valaisiana* (*ospA*), *Bo. lusitaniae* (*rpoB*), *Bo. spielmanii* (*fla*), *Bo. bissettii* (*rpoB*), *Bo. miyamotoi* (*glpQ*), *Bo. mayonii* (*fla*), *Bo. bavariensis* (*pyrG*), *Candidatus Neorickettsia mikurensis* (*groEL*), *Coxiella* (*C.*) spp. (*IS1111* and *icd*), *Ehrlichia* (*E*.) spp. (16S), *E. canis* (*gltA*), *Francisella* spp. (*tul*4 and *fopA*), *Hepatozoon* spp. (18S), *Rickettsia* (*R*.) spp. (*gltA*), *R. conorii* (ITS), *R. slovaca* (*ITS*), *R. massiliae* (ITS), *R. helvetica* (ITS), *R. aeschlimannii* (ITS), *R. felis* (*orfB*) and *Theileria* (*Th*.) spp. (18S) (see Supplementary Materials Table S2). A no-DNA (negative) control and a DNA-extraction-control to ensure the efficient amplification of ITS-2 and 16S from tick DNA were included in the run on each chip; also included were the DNA of *Escherichia* (*Es*.) *coli* (EDL933 strain) as a microorganism spike-control to ensure efficient amplification/detection using *Es. coli* (*eae*)-specific primers/probes in solution (see Supplementary Materials Table S2). The PCR results were validated (only when genus-level detection was achieved) using conventional PCR targeting in the 18S rRNA region [42] (see Supplementary Materials Table S1) and the resultant amplicons were sequenced as described in Section 2.3.

### 2.5. Sequence and Phylogenetic Analyses

The sequences of *cox*1, 16S and ITS-2 (ticks) and 18S (TBPs) obtained were examined for quality and then assembled using the denovo assembly function in Geneious Prime 2019.0.4 (http://www.geneious.com). Duplicate sequences were removed using the “find duplicates” function in Geneious and sequences unique to each locus were aligned using MUSCLE v.3.8.31 [43] within MEGA 7.0 [44]. The BLASTn function of the National Centre for Biotechnology Information (NCBI) (https://blast.ncbi.nlm.nih.gov/Blast.cgi) was used to match the identities of individual sequences; sequence identity matrices were established using BioEdit [45]. After verifying that all *cox*1 sequences had open reading frames, all unique nucleotide sequences were deposited into the GenBank database (Table 1). Reference sequences for individual loci (representing ticks or TBPs) were obtained from GenBank and aligned. Sequences obtained for ticks were trimmed to consensus lengths of 409 (16S), 549 (*cox*1) and 293 (ITS-2) bp; sequences obtained for TBPs were aligned over 478 bp (18S).

Phylogenetic analyses were performed on individual 16S, *cox*1 and ITS-2 sequence datasets using the Bayesian Inference (BI), Neighbor Joining (NJ) and Maximum Likelihood (ML) methods. The BI was conducted using the MrBayes plugin within Geneious [46] whereas NJ and ML analyses were performed using MEGA. The best-fit evolutionary models were estimated separately for individual sequence alignments (Tamura 3-parameter model [47] with gamma-distribution for 16S, Tamura-Nei model [48] with a proportion of invariable sites and gamma distribution for *cox*1 and Tamura 3-parameter model [47] with a proportion of invariable sites for ITS-2) using the Bayesian information criteria in jModelTest v.3.7 [49]. The nodal support in NJ and ML trees was tested through bootstrap analyses (10,000 replicates). The posterior probabilities of BI analyses were calculated for 2,000,000 generations (ngen = 2,000,000), saving every 100th tree (samplefreq = 100). Phylogenetic trees of ticks were rooted using *Argas* (*Ar.*) *persicus* (16S: KJ13358, *cox*1: FN394341) and *Haemaphysalis* (*Hs.*) *longicornis* (ITS-2: HQ005301) as outgroups whereas those of TBPs were rooted using *B. bigemina* (18S: KF112076).

### 2.6. Statistical Analyses

Results obtained from microfluidic PCR-based analysis/testing were analyzed using GraphPad 5 Prism (GraphPad software Inc. La Jolla, CA, USA). Chi-square and Fisher’s exact tests were used to compare the occurrence of microorganisms in different tick species from different hosts and tribal districts.

## 3. Results

### 3.1. Morphological Characterization of Ticks

The morphological examination of ticks (*n* = 104) revealed that the majority belonged to the genus *Rhipicephalus* (*Rh. haemaphysaloides*: *n* = 10; *Rh. turanicus*: *n* = 33; *Rh. microplus*: *n* = 15) followed by *Haemaphysalis* (*Hs. sulcata*: *n* = 26; *Hs. punctata*: *n* = 7) and *Hyalomma* (*Hy. anatolicum*: *n* = 13) (Table 1). *Rhipicephalus turanicus, Rh. microplus*, *Hs. sulcata* and *Hy. anatolicum* were present on both sheep and goats whereas *Hs. punctata* and *Rh. haemaphysaloides* occurred only on goats. *Rhipicephalus turanicus* was found in all five tribal districts whereas *Rh. microplus*, *Rh. haemaphysaloides* and *Hs. sulcata* were each found in three districts (i.e., Bajaur, Mohmand and North Waziristan; Khyber, Mohmand and North Waziristan; Bajaur, Khyber and North Waziristan, respectively). *Haemaphysalis punctata* and *Hy. anatolicum* occurred exclusively in two (Bajaur and North Waziristan) and one (Mohmand) districts, respectively (Table 1).

### 3.2. Sequence and Phylogenetic Analyses of Ticks

A total of 25 unique sequences of 16S (*n* = 12) and *cox*1 (*n* = 13) were obtained with most sequences representing *Rhipicephalus* (14) followed by *Haemaphysalis* (9) and *Hyalomma* (2). Partial ITS-2 unique sequences (*n* = 4) were also obtained for *Hyalomma* (*n* = 1) and *Rhipicephalus* (*n* = 3), which confirmed the identity of closely related species within these two genera. Intraspecific pairwise comparisons revealed the highest nucleotide differences within *Rh. haemaphysaloides* (*cox*1: 0.2–7.6%) followed by *Hs. sulcata* (16S: 0.3–1.5% and *cox*1: 0.8–1.8%, respectively) and *Rh. microplus* (0.3–0.5% and 0.2%, respectively) (Supplementary Materials Tables S3–S5).

**Figure 2:**
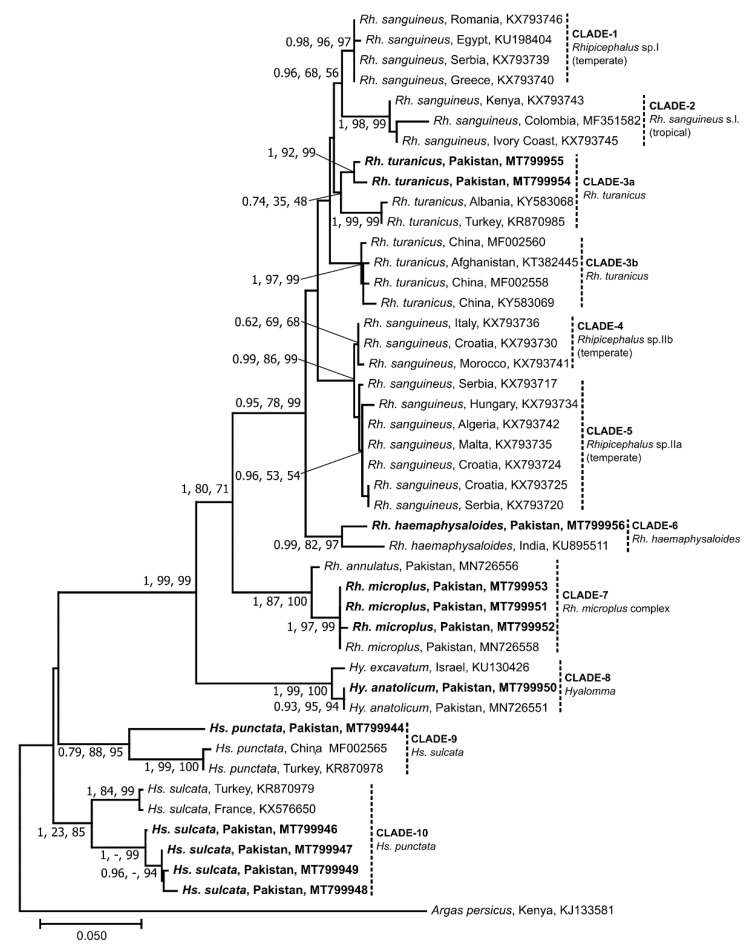
Phylogenetic relationships of partial 16S rRNA sequences of ticks collected from small ruminants in the FATA of Pakistan. Phylogenies were inferred using Neighbor Joining (NJ, this tree), Maximum Likelihood (ML) and Bayesian Inference (BI) methods. Sequences determined herein are shown in bold. The country of origin and GenBank accession numbers for each sequence are also provided. Nodal support values are presented as a posterior probability for BI (left) followed by bootstrap values for ML and NJ. The scale-bar indicates the number of inferred substitutions per site.

**Figure 3:**
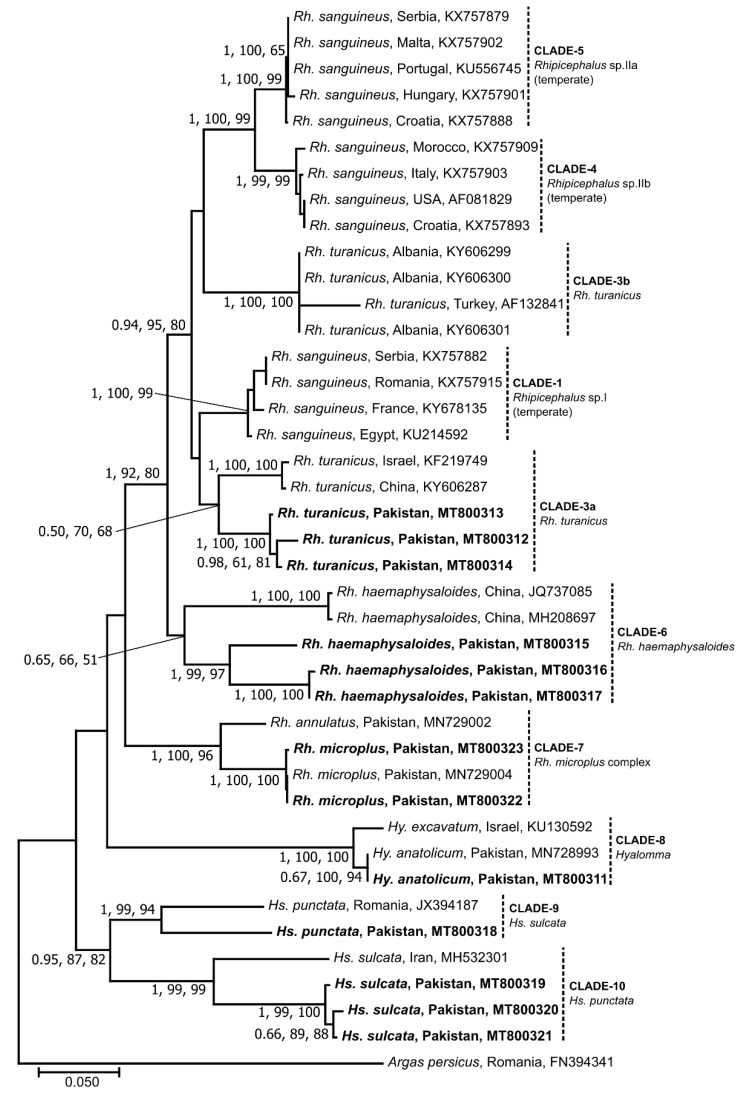
Phylogenetic relationships of partial *cox*1 sequences of ticks collected from small ruminants in the FATA of Pakistan. Phylogenies were inferred using Neighbor Joining (NJ, this tree), Maximum Likelihood (ML) and Bayesian Inference (BI) methods. Sequences determined herein are shown in bold. The country of origin and GenBank accession numbers for each sequence are also provided. Nodal support values are presented as a posterior probability for BI (left) followed by bootstrap values for ML and NJ. The scale-bar indicates the number of inferred substitutions per site.

**Figure 4:**
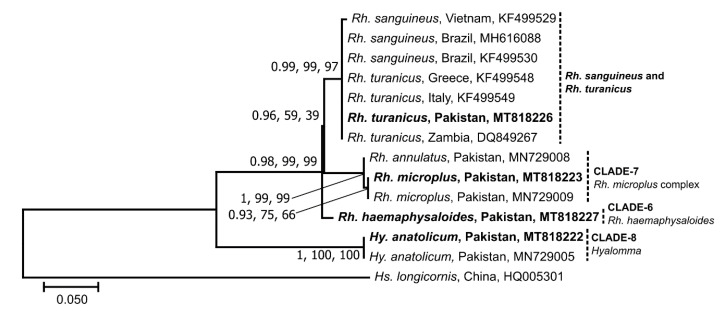
Phylogenetic relationships of partial sequences of the second internal transcribed spacer of the nuclear ribosomal DNA of *Rhipicephalus* and *Hyalomma* ticks collected from small ruminants in the FATA of Pakistan. Phylogenies were inferred using Neighbor Joining (NJ, this tree), Maximum Likelihood (ML) and Bayesian Inference (BI) methods. Sequences determined herein are shown in bold. The country of origin and GenBank accession numbers for each sequence are also provided. Nodal support values are presented as a posterior probability for BI (left) followed by bootstrap values for ML and NJ. The scale-bar indicates the number of inferred substitutions per site.

Separate phylogenetic analyses of 16S, *cox*1 or ITS-2 sequence data sets using the BI, ML and NJ methods produced trees with similar topologies; hence, only NJ trees were presented along with bootstrap and posterior probability (PP) support values for NJ and ML and BI, respectively (Figure 2, Figure 3 and Figure 4). For both 16S and *cox*1, the groupings were similar with a few minor differences. The sequences determined in this study grouped into six clades (clade-3a, 6–10) (Figure 2 and Figure 3). For 16S and *cox*1, clade-3a consisted of two (GenBank accession nos. MT799954 and MT799955) and three (GenBank: MT800312–MT800314) sequences, respectively, which grouped together with previously published sequences of *Rh. turanicus* from Albania, Turkey, China and Israel with a weak nodal support (16S: posterior probabilities for BI = 0.74; bootstrap for ML = 35%; bootstrap for NJ = 48%; *cox*1: 0.50, 70%, 68%) (Figure 2 and Figure 3). Clade-6 comprised one and three sequences of 16S (MT799956) and *cox*1 (GenBank: MT800315–MT800317), respectively, which clustered with *Rh. haemaphysaloides* from India and China with weak to strong statistical support (16S: 0.99, 82%, 97%; *cox*1: 0.65, 66%, 51%). Within clade-7, three and two sequences of 16S (GenBank: MT799951–MT799953) and *cox*1 (GenBank: MT800322 and MT800323), respectively, clustered with *Rh. microplus* sequences from Pakistan with strong nodal support values (16S: 1, 97%, 99%; *cox*1: 1, 100%, 100%) (Figure 2 and Figure 3). Clade-8 contained one sequence each of 16S (GenBank: MT799950) and *cox*1 (GenBank: MT800311), which grouped with *Hy. anatolicum* from Pakistan with weak to strong statistical support (16S: 0.93, 95%, 94%; *cox*1: 0.67, 100%, 94%) (Figure 2 and Figure 3). Clade-9 also consisted of one sequence each for 16S (GenBank: MT799944) and *cox*1 (GenBank: MT800318), which grouped with *Hs. punctata* sequences from China, Turkey and Romania with weak to strong nodal support (16S: 0.79, 88%, 95%; *cox*1: 1, 99%, 94%). Clade-10 comprised four and three sequences of 16S (GenBank: MT799946–MT799949) and *cox*1 (GenBank: MT800319–MT800321), respectively, which clustered with *Hs. sulcata* sequences from France, Turkey and Iran with variable nodal support (16S: 1, 23%, 85%; *cox*1: 1, 99%, 99%) (Figure 2 and Figure 3).

The relationships of ITS-2 sequences (GenBank: MT818222 and MT818223) were consistent with those obtained using 16S and *cox*1 data (Figure 4). However, the *Rh. turanicus* sequence (GenBank: MT818226) clustered with *Rh. turanicus* and *Rh. sanguineus* sequences from Zambia, Italy, Brazil and Vietnam with strong nodal support (0.99, 99%, 97%). Moreover, no reference ITS-2 sequence was available for *Rh. haemaphysaloides* and the sequence of a specimen (GenBank: MT818227), which grouped with *Rh. haemaphysaloides* in 16S and *cox*1 phylogenies (Figure 2 and Figure 3). The corresponding ITS-2 sequence grouped outside of the *Rhipicephalus* group with strong nodal support (1, 99%, 99%) (Figure 4).

### 3.3. Diversity of Microorganisms in Ticks

DNA of at least one of 11 microorganisms was detected in a total of 39 of 54 (72.2%) ticks tested (Table 2). *Rickettsia massiliae* was the most commonly detected pathogen (42.6%) followed by *Theileria* spp. (33.3%), *A. ovis* and *R. slovaca* (25.9%), *A. centrale* (9.3%), *Ehrlichia* spp., *R. conorii* and *R. aeschlimannii* (5.6%) and *A. marginale* (1.9%) (Table 3). Furthermore, endosymbionts including *Francisella*-like and *Coxiella*-like were also detected in 22.2% and 7.4% of ticks, respectively (Table 3).

**Figure 5:**
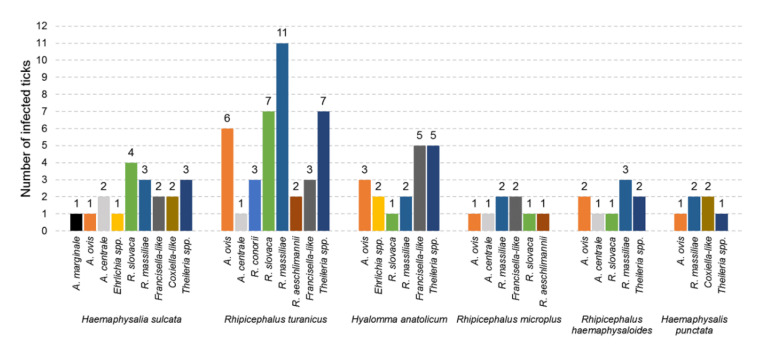
Number of ticks infected with microorganisms from small ruminants in the FATA of Pakistan. The species of tick is shown at the bottom while the number of individual species of ticks infected with the microorganism species (*x* axis) is shown above each bar (*y* axis) of the chart.

The occurrence of microorganisms varied significantly across different districts (*x^2^* = 61.5, *df* = 4, *P* < 0.0001) (Bajaur, 41.7%; Khyber, 75%; Mohmand, 86%; Orakzai, 75%; North Waziristan, 83%) as well as between ticks (*P* < 0.0001) collected from sheep (56.5%) and goats (83.9%) (Table 2 and Table 3). A significant variation (*x^2^* = 90.33, *df* = 5, *P* < 0.0001) was recorded in the prevalence of microbes in different tick species; it was highest in *Hy. anatolicum* and *Rh. haemaphysaloides* (100%) followed by *Hs. punctata* (75%), *Rh. turanicus* (66.7%), *Hs. sulcata* (64.3%) and *Rh. microplus* (62.5%) (Table 2, Figure 5). The most diversity of microbes was detected within *Hs. sulcata* (9 of 11 microorganisms) followed by *Rh. turanicus* (8), *Hy. anatolicum* and *Rh. microplus* (6), *Rh. haemaphysaloides* (5) and *Hs. punctata* (4) (Figure 5).

### 3.4. Co-Occurrence of Microorganisms in Ticks

Of the 39 ticks found positive for microbes, DNA of one or more microorganisms was present in 11 (28.2%) and 28 (71.8%) ticks, respectively (Table 4). DNA of two, three, four, five or seven microorganisms was found in 28.6%, 39.2%, 25%, 3.6% and 3.6% ticks, respectively (Table 4). DNA of six microorganisms (*A. ovis*, *A. centrale*, *R. slovaca*, *R. massiliae*, *Francisella*-like and *Theileria* spp.) was present either as single or a mixed infection whereas DNA of the other five microbes was found only as mixed infections (Table 4). *Rh. turanicus* ticks were positive for a maximum of seven microorganisms followed by *Hs. sulcata*, *Hy. anatolicum*, *Rh. haemaphysaloides* and *Rh. Microplus*, which were positive for four and *Hs. punctata* for three (data not shown).

### 3.5. Genetic Relationship of Selected Microorganisms

**Figure 6:**
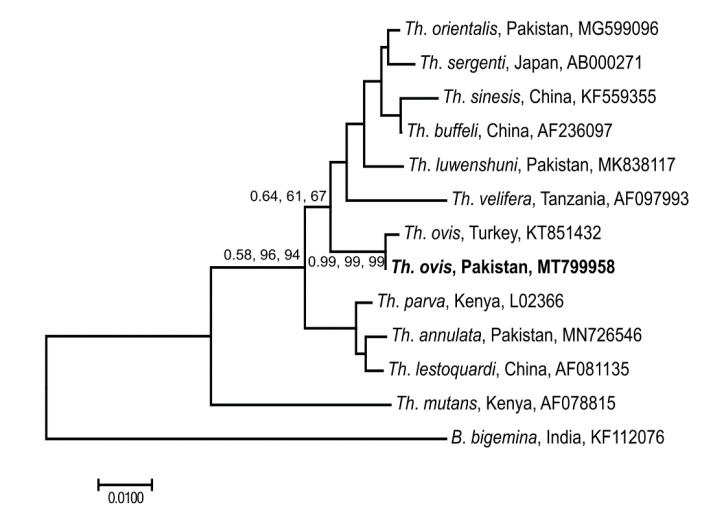
Phylogenetic relationship of 18S sequences of *Theileria* spp. from ticks of small ruminants in the FATA of Pakistan. Phylogenies were inferred using Neighbor Joining (NJ, this tree), Maximum Likelihood (ML) and Bayesian Inference (BI) methods. Sequences determined herein are shown in bold. The country of origin and GenBank accession numbers for each sequence are also provided. Nodal support values are presented as a posterior probability for BI (left) followed by bootstrap values for ML and NJ. The scale-bar indicates the number of inferred substitutions per site.

Phylogenetic analyses (using BI, ML and NJ) of the 18S sequence data for piroplasms determined herein (GenBank: MT799958) confirmed the presence of *Th. ovis* as it grouped with the reference sequence of *Th. ovis* from Turkey (GenBank: KT851432) with strong nodal support (0.99, 99%, 99%) (Figure 6).

## 4. Discussion

This study provides the first insight into the molecular diversity of ticks, TBPs and endosymbionts in ticks from small ruminants in the FATA, Pakistan. The occurrence of *Hyalomma* and *Rhipicephalus* species characterized in our study is consistent with what has been reported previously from small and large ruminants in selected areas of Pakistan [8,15,50]. However, this study provides the first genetic evidence for *Hs. sulcata*, *Hs. punctata*, *Rh. haemaphysaloides* and *Rh. turanicus* from Pakistan. 

Within the *Rh. sanguineus* group (clades 1–5), the sequences of *Rh. turanicus* determined here (clade-3a) clustered with the sequences belonging to the temperate lineage of *Rh. sanguineus* (clade-1) (Figure 2 and Figure 3) indicating a close similarity between the two species. The NCBI blast results also supported these findings as two of the 16S sequences determined here (GenBank: MT799954 and MT799955) were similar (96–97.2%) to those of *Rh. sanguineus* (GenBank: KR870984) and *Rh. turanicus* (GenBank: KR870985) from Turkey (data not shown). Likewise, three *cox*1 sequences (GenBank: MT800312–MT800314) were similar (90.5–93.9%) to those of *Rh. sanguineus* (GenBank: MF426015) and *Rh. turanicus* (GenBank: MN853166) from Portugal and Kazakhstan, respectively (data not shown). The taxonomic classification of *Rh. turanicus* has been recently studied by Bakkes et al. [51] who refuted the monophyletic nature of *Rh. turanicus* and proposed a new species, i.e., *Rh. africanus* n sp. Moreover, the authors also provided evidence of the existence of two lineages corresponding to southern Europe and the Middle East/Asia with differing climates. We also found a similar pattern here as *Rh. turanicus* sequences (from this study and references) clustered into two distinct clades (3a and 3b) (Figure 2 and Figure 3). These two clades may represent two separate species (cf. [51]). For *Rh. haemaphysaloides*, the genetic differences (0.2–7.6%) in *cox*1 sequences inferred here suggest the existence of two distinct lineages or even distinct species as their genetic similarity is < 95%, the value generally considered to be the threshold of conspecificity for these genes in ticks [52,53,54,55,56]. However, due to limited genetic data being available for this tick species, it is challenging to define a threshold for species delineation.

This study also provides the first molecular evidence for *Hs. sulcata* and *Hs. punctata* in Pakistan. The molecular-phylogenetic analyses of *Haemaphysalis* sequences confirmed and supported morphological characterization. However, the NCBI blast results demonstrated considerably large nucleotide differences from previously reported sequences of the same species (*Hs. sulcata*: 16S; 4.7–5.5%, *cox*1; 10.4–10.7%, *Hs. punctata*: 16S; 6.8%, *cox*1; 10.7%) from Turkey and Iran (*Hs. sulcata*) and China and Romania (*Hs. punctata*) (data not shown). These large differences could be partly due to the limited availability of gene sequences of these ticks. Future studies should focus on the morphological and genetic characterization of *Haemaphysalis* ticks collected from different climatic zones of Pakistan. Furthermore, additional genetic markers such as the elucidation of complete mitochondrial genomes [56,57] would allow the phylogeny of *Haemaphysalis* and other ticks to be resolved.

In this study, microfluidic real-time PCR-based screening of ticks demonstrated a higher prevalence of microorganisms (72.2%) as well as a higher percentage of ticks (71.8%) testing positive for multiple microorganisms (Table 3 and Table 4). The results were validated by conventional PCR for microorganisms whose specific identification was not achieved. However, genetic characterization was not successful for some microorganisms (such as the *Ehrlichia* species) due to low cycle threshold (Ct) values. Moreover, this study does not establish the mammalian- or tick-origin of detected microorganisms since ticks were collected while feeding on their hosts (i.e., goats and sheep). In addition, the detection of DNA of multiple microorganisms in several ticks does not imply the co-transmission to their hosts. Furthermore, this study does not provide estimates for the prevalence and distribution of different tick species in small ruminants from the FATA as ticks were collected from a smaller population. However, the prevalence of microorganisms was significantly higher in ticks collected from goats (83.9%) compared with those from sheep (56.5%) as shown in Table 3. Although there have been reports of higher prevalences of ticks [15] and internal parasites [58] in goats compared with sheep, most reports on the prevalence of haemoparasites in small ruminants from Pakistan and elsewhere are contrary to this finding [11,59,60,61,62]. For example, Iqbal et al. [11] reported 32 and a 5% prevalence of piroplasms in sheep and goats from the Punjab and KPK provinces of Pakistan, respectively. Likewise, Azmi et al. [59] and Rjeibi et al. [61] found higher prevalences of *Theileria* and piroplasms in sheep compared with goats from Palestine and Tunisia, respectively. Further molecular testing would be required to establish such differences, if any, as a smaller number of ticks were tested in the present study.

*Francisella*-like and *Coxiella*-like endosymbionts detected in this study are non-pathogenic mutualistic and/or commensal microbes, which play a key role in the tick’s developmental process and pathogen transmission [63,64]. We detected a higher prevalence of DNA of *R. massiliae* and *R. slovaca* in ticks in all five districts with a low prevalence of *R. aeschlimannii* (Bajaur and Mohmand) and *R. conorii* (Khyber and Mohmand) (see Table 3). All of these rickettsiae belong to the spotted fever group (SFG) and, thus, have zoonotic potential. The significance and risk of infections of SFG rickettsiae are higher in Asian countries where surveillance and diagnostic facilities are limited and new cases of rickettsial infections are increasing [65]. Due to a large influx of livestock and immigrants during the Afghan War, it is possible that new potential pathogens might have been imported into this region [7]. Moreover, most of the families in the FATA are living with their animals and there is a general lack of awareness about TBDs of veterinary and public health importance [7]. Our findings and previous reports of rickettsial species from ticks in Pakistan [28] highlight the need to establish a diagnostic surveillance system for zoonotic rickettsial pathogens in this country.

A higher prevalence of *Theileria* spp. (33.3%) was also found in ticks from all five districts and the molecular characterization of piroplasms revealed that they belonged to *Th. ovis*. However, it is not possible to exclude the possibility of another caprine/ovine *Theileria* species as *Th. lestoquardi* has been reported from small ruminants in Pakistan such as by Riaz et al. [16]. *Anaplasma ovis* was detected in 25.9% of all tick species identified herein and it is most frequently associated with anaplasmosis in small ruminants worldwide [64]. However, most of these cases are subclinical infections with a low-grade fever [66]. More recently, a variant of *A. ovis* has also been associated with human infection in Cyprus [67]. However, due to the lack of a proper diagnostic and surveillance system in Pakistan it is difficult to ascertain the economic losses and zoonotic threat due to this and other TBPs detected in this study.

Finding ticks that carry DNA of up to seven microorganisms of veterinary and medical significance indicates the level of risk associated with tick infestation to animals as well as humans. Previously, a similar level of co-occurrence of endosymbionts (i.e., *Francisella*-like and *Coxiella*-like) and pathogens (belonging to *Anaplasma*, *Babesia*, *Bartonella*, *Borrelia*, *Ehrlichia*, *Hepatozoon*, *Rickettsia* and *Theileria* genera) in bovine ticks was reported from the Punjab and Sindh provinces of Pakistan [28]. Similarly, microbiome analyses of *Dermacentor silvarum* and *Ixodes persulcatus* ticks from China revealed the presence of up to 29 and 373 bacterial genera, respectively, belonging to endosymbionts and pathogens [68,69]. These high levels of co-occurrence of microorganisms in ticks encourage the large-scale implementation of such high-throughput tools in resource-scarce settings where routine surveillance facilities are not accessible.

## 5. Conclusions

This study provides the first molecular insights into ticks, TBPs and endosymbionts of ticks from small ruminants of the FATA, Pakistan. Findings of this study demonstrate that multiple species of the *Rhipicephalus* group exist that are not distinguishable based on morphological data alone. Furthermore, ticks of small ruminants from the FATA carry multiple microorganisms of a veterinary and medical health significance. This study highlights the need to explore further tick and TBPs of livestock and wildlife species in this region of the country to guide the development of control measures and extension programs for farmers.

## Figures and Tables

**Table 1:** Details of ticks of small ruminants from the FATA of Pakistan used in this study. GenBank accession numbers are also provided for unique 16S, *cox*1 and ITS-2 sequences.

Tribal Districts (Coordinates)	Host (Number)	Tick Species (Based on Microscopy)	No. of Ticks	GenBank Accession Numbers (Only Unique Sequences)		
				16S	*cox*1	ITS-2
Bajaur (34.856902° N, 71.429936° E)	Sheep (*n* = 5)	*Haemaphysalis sulcata*	4	MT799946	MT800319	-
		*Rhipicephalus microplus*	6	MT799951	MT800322	-
		*Rhipicephalus turanicus*	3	-	-	-
	Goat (*n* = 7)	*Haemaphysalis punctata*	3	MT799944	MT800318	-
		*Hs*. *sulcata*	6	MT799946	MT800319	-
				MT799947	MT800320	-
		*Rh*. *microplus*	2	-	-	-
		*Rh*. *turanicus*	4	-	-	-
Khyber (33.940548° N, 71.049777° E)	Sheep (*n* = 5)	*Hs*. *sulcata*	5	-	-	-
		*Rh*. *turanicus*	3	-	-	-
	Goat (*n* = 7)	*Hs*. *sulcata*	5	MT799948	MT800320	-
		*Rh*. *turanicus*	4	MT799955	MT800313	-
		*Rhipicephalus haemaphysaloides*	5	MT799956	MT800316	MT818227
				MT799956	MT800315	MT818227
Mohmand (34.535595° N, 71.287421° E)	Sheep (*n* = 6)	*Hyalomma anatolicum*	6	MT799950	MT800311	MT818222
		*Rh*. *turanicus*	5	MT799954	MT800312	MT818226
				MT799954	MT800314	MT818226
	Goat (*n* = 8)	*Hy*. *anatolicum*	7	MT799950	MT800311	MT818222
		*Rh*. *microplus*	1	-	-	-
		*Rh*. *turanicus*	5	-	-	-
		*Rh. haemaphysaloides*	3	MT799956	-	-
Orakzai (33.667137° N, 70.95468° E)	Sheep (*n* = 2)	*Rh*. *turanicus*	4	-	-	-
	Goat (*n* = 2)	*Rh*. *turanicus*	3	-	-	-
North Waziristan (32.320237° N, 69.859741° E)	Sheep (*n* = 5)	*Hs*. *sulcata*	6	MT799949	MT800321	-
		*Rh*. *microplus*	2	MT799952	MT800323	MT818223
	Goat (*n* = 7)	*Hs*. *punctata*	4	-	-	-
		*Rh*. *microplus*	4	MT799953	MT800322	MT818223
		*Rh*. *turanicus*	2	-	-	-
		*Rh*. *haemaphysaloides*	2	MT799956	MT800317	MT818227
Total	54		104			

**Table 2:** Occurrence of various microorganisms in six tick species of small ruminants from the FATA of Pakistan.

Tick Species	District	Host	No. of Ticks Tested	No. of Ticks Infected	Microorganisms Detected
*Haemaphysalis punctata*	Bajaur, North Waziristan	Goat	4	3	*Anaplasma ovis*, *Rickettsia massiliae*, *Coxiella*-like, *Theileria* spp.
*Hs. sulcata*	Bajaur, Khyber, North Waziristan	Sheep	8	4	*A. marginale*, *A. ovis*, *A. centrale, R. slovaca*, *R. massiliae, Francisella*-like, *Coxiella*-like, *Theileria* spp.
		Goat	6	5	*A. centrale, Ehrlichia spp., R. slovaca, R. massiliae, Francisella*-like, *Coxiella*-like, *Theileria* spp.
*Hyalomma anatolicum*	Mohmand	Sheep	3	3	*A. ovis*, *Ehrlichia* spp., *R. slovaca, R. massiliae, Francisella*-like, *Theileria* spp.
		Goat	3	3	*A. ovis*, *R. massiliae, Francisella*-like, *Theileria* spp.
*Rhipicephalus microplus*	Bajaur, Mohmand, North Waziristan	Sheep	4	2	*A. ovis*, *Francisella*-like, *R. aeschlimannii, R. massiliae, R. slovaca*
		Goat	4	3	*A. centrale, R. massiliae, Francisella*-like
*Rh. turanicus*	Bajaur, Khyber, Orakzai, Mohmand, North Waziristan	Sheep	8	4	*A. ovis, R. conorii, R. slovaca, R. massiliae, R. aeschlimannii, Francisella*-like, *Theileria* spp.
		Goat	10	8	*A. ovis, A. centrale, R. conorii, R. slovaca, R. massiliae, R. aeschlimannii, Francisella*-like, *Theileria* spp.
*Rh. haemaphysaloides*	Khyber, Mohmand, North Waziristan	Goat	4	4	*A. ovis, A. centrale, R. slovaca, R. massiliae, Theileria* spp.
Total			54	39	

**Table 3:** Prevalence of microorganisms in ticks of small ruminants in the FATA of Pakistan.

Microorganisms	Bajaur		Khyber		Mohmand		Orakzai		North Waziristan		% Prevalence (Proportion)
	Sheep (*n* = 5)	Goat (*n* = 7)	Sheep (*n* = 5)	Goat (*n* = 7)	Sheep (*n* = 6)	Goat (*n* = 8)	Sheep (*n* = 2)	Goat (*n* = 2)	Sheep (*n* = 5)	Goat (*n* = 7)	
*Anaplasma marginale*	-	-	1	-	-	-	-	-	-	-	1.9 (1/54)
*A. ovis*	1	-	1	1	3	4	1	-	1	2	25.9 (14/54)
*A. centrale*	-	1	1	-	-	1	-	-	-	2	9.3 (5/54)
*Ehrlichia* spp.	-	-	-	1	2	-	-	-	-	-	5.6 (3/54)
*Rickettsia conorii*	-	-	-	1	1	1	-	-	-	-	5.6 (3/54)
*R. slovaca*	1	2	-	3	3	1	1	2	1	-	25.9 (14/54)
*R. massiliae*	1	1	2	4	3	4	1	2	1	4	42.6 (23/54)
*R. aeschlimannii*	1	-	-	-	1	1	-	-	-	-	5.6 (3/54)
*Francisella-*like	2	-	-	1	4	3	1	1	-	-	22.2 (12/54)
*Coxiella-*like	-	-	-	1	-	-	-	-	1	2	7.4 (4/54)
*Theileria* spp.	1	1	2	1	3	5	1	2	-	2	33.3 (18/54)
Total	7	5	7	13	20	20	5	7	4	12	

**Table 4:** Single and mixed presence of DNA of microorganisms in ticks of small ruminants in the FATA of Pakistan.

Microorganisms	Number of Ticks Positive for Single and Mixed Infections					
	Bajaur (*n* = 12)	Khyber (*n* = 12)	Mohmand (*n* = 14)	Orakzai (*n* = 4)	North Waziristan (*n* = 12)	Total
**Single**						
*Anaplasma ovis*	-	-	-	-	1	1
*A. centrale*	-	-	-	-	1	1
*Rickettsia slovaca*	1	1	-	-	1	3
*R. massiliae*	-	1	-	-	2	3
*Francisella*-like	-	-	1	-	-	1
*Theileria* spp.	1	1	-	-	-	2
**Double**						
*A. centrale*, *R. massiliae*	-	-	-	-	1	1
*A. ovis*, *Theileria* spp.	-	-	1	-	1	2
*A. ovis*, *R. massiliae*.	-	1	-	-		1
*A. ovis*, *Coxiella*-like	-	-	-	-	1	1
*Francisella*-like, *Theileria* spp.	-	-	1	-	-	1
*R. massiliae*, *R. slovaca*	-	1	-	-	-	1
*R. massiliae*, *Coxiella*-like	-	-	-	-	1	1
**Triple**						
*A. ovis*, *R. massiliae*, *Theileria* spp.	-	1	1	-	-	2
*A. ovis*, *R. massiliae*, *R. slovaca*	-	-	1	-	-	1
*A. ovis*, *Francisella*-like, *Theileria* spp.	1	-	-	-	-	1
*A. centrale*, *R. massiliae*, *R. slovaca*	1	-	-	-	-	1
*Coxiella*-like, *Ehrlichia* spp., *Francisella*-like,	-	1	-	-	-	1
*Coxiella*-like, *R. massiliae*, *Theileria* spp.	-	-	-	-	1	1
*Ehrlichia* spp., *Francisella*-like, *Theileria* spp.	-	-	1	-	-	1
*Francisella*-like, *R. massiliae*, *R. slovaca*	-	-	1	-	-	1
*R. conorii*, *R. massiliae*, *R. slovaca*	-	1	-	-	-	1
*R. massiliae*, *R. slovaca*, *Theileria* spp.	-	-	-	1	-	1
**Quadruple**						
*A. centrale*, *A. marginale*, *R. massiliae*, *Theileria* spp.	-	1	-	-	-	1
*A. centrale*, *A. ovis*, *R. massiliae*, *Theileria* spp.	-		1	-	-	1
*A. ovis*, *Ehrlichia* spp., *Francisella*-like, *Theileria* spp.	-	-	1	-	-	1
*A. ovis*, *R. massiliae*, *Francisella*-like, *Theileria* spp.	-	-	1	-	-	1
*Francisella*-like, *R. aeschlimannii*, *R. massiliae*, *R. slovaca*	1	-	-	-	-	1
*Francisella*-like, *R. massiliae*, *R. slovaca*, *Theileria* spp.	-	-	-	1	-	1
*R. aeschlimannii*, *R. conorii*, *R. massiliae*, *R. slovaca*	-		1	-	-	1
**Quintuple**						
*A. ovis*, *Francisella*-like, *R. massiliae*, *R. slovaca*, *Theileria* spp.	-	-	-	1	-	1
**Septuple**						
*A. ovis*, *Francisella*-like, *R. aeschlimannii*, *R. conorii*, *R. massiliae*, *R. slovaca*, *Theileria* spp.	-	-	1	-	-	1
Total	5	9	12	3	10	39

## Data Availability

The datasets supporting the conclusion of this article are included within the article and supplementary material files. Nucleotide sequences reported in this article are available via GenBank.

## Supplementary Materials

The following are available online at https://www.mdpi.com/2076-2607/8/9/1428/s1, Table S1: PCR primers and thermocycling conditions used in this study, Table S2: List of primers and probes used in this study for the identification of microorganisms and ticks, Table S3: Pairwise comparisons of 16S nucleotide sequences (aligned over 429 bp) of ticks determined herein. Nucleotide similarity and percentage differences are given above and below the diagonal, respectively, Table S4: Pairwise comparisons of *cox*1 nucleotide sequences (aligned over 673 bp) of ticks determined herein. Nucleotide similarity and percentage differences are given above and below the diagonal, respectively, Table S5: Pairwise comparisons of ITS-2 nucleotide sequences (aligned over 273 bp) of ticks belonging to *Rhipicephalus* and *Hyalomma* genera determined herein. Nucleotide similarity and percentage differences are given above and below the diagonal, respectively.
