# Supplementary material for: An Assessment of the Molecular Diversity of Ticks and Tick-Borne Microorganisms of Small Ruminants in Pakistan
Source: Microorganisms. 2020 Sep 17;8(9):1428. doi: 10.3390/microorganisms8091428 (PMC7563897; doi:10.3390/microorganisms8091428)
Supplement: Supplementary file 1 [file microorganisms-08-01428-s001.pdf]

Table S1

PCR primers and thermocycling conditions used in this study.

**Target region**	**Primers**	**PCR conditions**	**Amplicon size (base pairs)**	**Reference**
16S for ticks	Forward: 16S+1 (5'-CTGCTCAATGATTTTTTAAATTGCTGTGG-3')<br>Reverse: 16S-1 (5'-CCGGTCTGAACTCAGATCAAGT-3')	Initial denaturation: 95 °C, 4min<br>35 cycles of<br>Denaturation: 95 °C, 1min<br>Annealing: 50 °C, 1min<br>Extension: 68 °C, 1min<br>Final extension:68 °C, 10min	460	[25]
*cox*1 for ticks	Forward: LCO1490 (5'- GGTCAACAAATCATAAAGATATTGG-3')<br>Reverse: HCO2198 (5'-TAAACTTCAGGGTGACCAAAAAATCA-3')	Initial denaturation: 95 °C, 5min<br>40 cycles of<br>Denaturation: 95 °C, 45sec<br>Annealing 50 °C, 45sec<br>Extension: 72 °C, 1min<br>Final extension: 72 °C, 5min	710	[40]
ITS-2 for ticks	Forward: RIB-8 (5'-GTCGTAGTCCGCCGTC-3')<br>Reverse: RIB-11 (5'-GAGTACGACGCCCTACC-3')	Initial denaturation: 94 °C, 5min<br>35 cycles of<br>Denaturation: 94 °C, 30sec<br>Annealing: 62 °C, 15sec<br>Extension: 72 °C, 15sec<br>Final extension: 72 °C, 5min	280	[41]
18S for piroplasms	Forward: (5'-GAGGTAGTGACAAGAAATAACAATA-3')<br>Reverse: (5'-TCTTCGATCCCCTAACTTTC-3')	Initial denaturation: 95 °C, 5min<br>35 cycles of<br>Denaturation: 95 °C, 30sec<br>Annealing: 57 °C, 20 sec<br>Extension: 72 °C, 1 min<br>Final extension: 72 °C, 5min	500	[42]

Table S2

List of primers and probes used in this study for identification of microorganisms and ticks.

Species	Target	Name	Sequence	Length (base pair)
Anaplasma marginale	*msp1*	An_ma_msp1_F	CAGGCTTCAAGCGTACAGTG	85
		An_ma_msp1_R	GATATCTGTGCCTGGCCTTC	
		An_ma_msp1_P	ATGAAAGCCTGGAGATGTTAGACCGAG	
Anaplasma platys	*groEL*	An_pla_groEL_F	TTCTGCCGATCCTTGAAAACG	75
		An_pla_groEL_R	CTTCTCCTTCTACATCCTCAG	
		An_pla_groEL_P	TTGCTAGATCCGGCAGGCCTCTGC	
Anaplasma ovis	*msp4*	An_ov_msp4_F	TCATTCGACATGCGTGAGTCA	92
		An_ov_msp4_R	TTTGCTGGCGCACTCACATC	
		An_ov_msp4_P	AGCAGAGAGACCTCGTATGTTAGAGGC	
Anaplasma centrale	*groEL*	An_cen_groEL_F	AGCTGCCCTGCTATACACG	79
		An_cen_groEL_R	GATGTTGATGCCCAATTGCTC	
		An_cen_groEL_P	CTTGCATCTCTAGACGAGGTAAAGGGG	
Anaplasma phagocytophilum	*msp2*	An_ph_msp2_F	GCTATGGAAGGCAGTGTTGG	77
		An_ph_msp2_R	GTCTTGAAGCGCTCGTAACC	
		An_ph_msp2_P	AATCTCAAGCTCAACCCTGGCACCAC	
Anaplasma bovis	groEL	An_bo_groEL_F	CTGATAGCTACAGTTAAGCCC	73
		An_bo_groEL_R	AGGTGCTGTTGGATGTACTGCTGGACC	
		An_bo_groEL_P	TCATTCGACATGCGTGAGTCA	
Anaplasma spp.	16S rRNA	Ana_spp_16S_F	CTTAGGGTTGTAAAACTCTTTCAG	160
		Ana_spp_16S_R	CTTTAACTTACCAAACCGCCTAC	
		Ana_spp_16S_P	ATGCCCTTTACGCCCAATAATTCCGAACA	
Apicomplexa	18S rRNA	Apic_18S_F	TGAACGAGGAATGCCTAGTATG	104
		Apic_18S_R	CACCGGATCACTCGATCGG	
		Apic_18S_S	TAGGAGCGACGGGCGGTGTGTAC	
Babesia divergens	*hsp70*	Bab_di_hsp70_F	CTCATTGGTGACGCCGCTA	83
		Bab_di_hsp70_R	CTCCTCCCGATAAGCCTCTT	
		Bab_di_hsp70_P	AGAACCAGGAGGCCCGTAACCCAGA	

Babesia caballi	*Rap1*	Ba_cab_rap1_F	GTTGTTCGGCTGGGGCATC	94
		Ba_cab_rap1_R	CAGGCGACTGACGCTGTGT	
		Ba_cab_rap1_P	TCTGTCCCGATGTCAAGGGGCAGGT	
Babesia canis	18S rRNA	Ba_ca_RNA18S_F	TGGCCGTTCTTAGTTGGTGG	104
		Ba_ca_RNA18S_R	AGAAGCAACCGGAAACTCAAATA	
		Ba_ca_RNA18S_P	ACCGGCACTAGTTAGCAGGTTAAGGTC	
Babesia microti	*CCTeta*	Bab_mi_CCTeta_F	ACAATGGATTTTCCCCAGCAAAA	145
		Bab_mi_CCTeta_R	GCGACATTTCGGCAACTTATATA	
		Bab_mi_CCTeta_P	TACTCTGGTGCAATGAGCGTATGGGTA	
Babesia bovis	*CCTeta*	Ba_bo_CCTeta_F	GCCAAGTAGTGGTAGACTGTA	100
		Ba_bo_CCTeta_R	GCTCCGTCATTGGTTATGGTA	
		Ba_bo_CCTeta_P	TAAAGACAACACTGGGTCCGCGTGG	
Babesia bigemina	18S rRNA	Ba_big_RNA18S_F	ATTCCGTTAACGAACGAGACC	99
		Ba_big_RNA18S_R	TTCCCCCACGCTTGAAGCA	
		Ba_big_RNA18S_P	CAGGAGTCCCTCTAAGAAGCAAACGAG	
Babesia ovis	18S rRNA	Ba_ov_RNA18S_F	TCTGTGATGCCCTTAGATGTC	92
		Ba_ov_RNA18S_R	GCTGGTTACCCGCGCCTT	
		Ba_ov_RNA18S_P	TCGGAGCGGGGTCAACTCGATGCAT	
Babesia vulpes	*COX1*	Bab_vulpes_F	ACT TCC AGT GTT AAC AGC AGC	129
		Bab_vulpes_R	GGA TGT CCG AAG ACC CAG AA	
		Bab_vulpes_P	CTT CTG CAT GTT GCT GGC TGA TAG GCA	
Bartonella henselae	*pap31*	Bar_he_pap31_F	CCGCTGATCGCATTATGCCT	107
		Bar_he_pap31_R	AGCGATTTCTGCATCATCTGCT	
		Bar_he_pap31_P	ATGTTGCTGGTGGTGTTTCCTATGCAC	
Bartonella spp.	ssrA	Bart_spp_ssrA_F	CGTTATCGGGCTAAATGAGTAG	118
		Bart_spp_ssrA_R	ACCCCGCTTAAACCTGCGA	
		Bart_spp_ssrA_P	TTGCAAATGACAACTATGCGGAAGCACGTC	
Borrelia burgdorferi sensu stricto	*rpoB*	Bo_bu_rpoB_F	GCTTACTCACAAAAGGCGTCTT	83
		Bo_bu_rpoB_R	GCACATCTCTTACTTCAAATCCT	
		Bo_bu_rpoB_P	AATGCTCTTGGACCAGGAGGACTTTCA	
Borrelia garinii	*rpoB*	Bo_ga_rpoB_F	TGGCCGAACTTACCCACAAAA	88
		Bo_ga_rpoB_R	ACATCTCTTACTTCAAATCCTGC	
		Bo_ga_rpoB_P	TCTATCTCTTGAAAGTCCCCCTGGTCC	

Borrelia afzelii	*fla*	Bo_af_fla_F	GGAGCAAATCAAGATGAAGCAAT	116
		Bo_af_fla_R	TGAGCACCCTCTTGAACAGG	
		Bo_af_fla_P	TGCAGCCTGAGCAGCTTGAGCTCC	
Borrelia valaisiana	*ospA*	Bo_va_ospA_F	ACTCACAAATGACAGATGCTGAA	135
		Bo_va_ospA_R	GCTTGCTTAAAGTAACAGTACCT	
		Bo_va_ospA_P	TCCGCCTACAAGATTTCCTGGAAGCTT	
Borrelia miyamotoi	*glpQ*	B_miya_glpQ_F	CACGACCCAGAAATTGACACA	94
		B_miya_glpQ_R	GTGTGAAGTCAGTGGCGTAAT	
		B_miya_glpQ_P	TCGTCCGTTTTCTCTAGCTCGATTGGG	
Borrelia spielmanii	*fla*	Bo_spi_fla_F	ATCTATTTTCTGGTGAGGGAGC	71
		Bo_spi_fla_R	TCCTTCTTGTTGAGCACCTTC	
		Bo_spi_fla_P	TTGAACAGGCGCAGTCTGAGCAGCTT	
Borrelia lusitaniae	*rpoB*	Bo_lus_rpoB_F	CGAACTTACTCATAAAAGGCGTC	87
		Bo_lus_rpoB_R	TGGACGTCTCTTACTTCAAATCC	
		Bo_lus_rpoB_P	TTAATGCTCTCGGGCCTGGGGGACT	
Borrelia bissettii	*rpoB*	Bo_bi_rpoB_F	GCAACCAGTCAGCTTTCACAG	118
		Bo_bi_rpoB_R	CAAATCCTGCCCTATCCCTTG	
		Bo_bi_rpoB_P	AAAGTCCTCCCGGCCCAAGAGCATTAA	
Borrelia bavariensis	*pyrG*	Bo_bavar_pyrG_F	GTC TTT GGT TCA TGT TGG AGC	141
		Bo_bavar_pyrG_R	CCC TCA TAT CCT TTG CCT CC	
		Bo_bavar_pyrG_P	AAA GCC TCC GGG AAC AAT AAT GCC GTC AA	
Borrelia mayonii	*fla*	Bo_mayo_fla_F	ACA CAC CAT CAT CAC TTT CAG G	173
		Bo_mayo_fla_R	TTG AGC ACC TTC TTG AAC AGG	
		Bo_mayo_fla_P	CGC AAC CTG AGC AGT TTG AGT TCC CT	
Borrelia spp.	23S rRNA	Bo_bu_sl_23S_F	GAGTCTTAAAAGGGCGATTTAGT	73
		Bo_bu_sl_23S_R	CTTCAGCCTGGCCATAAATAG	
		Bo_bu_sl_23S_P	AGATGTGGTAGACCCGAAGCCGAGT	
Candidatus	*groEL*	Neo_mik_groEL_F	AGAGACATCATTCGCATTTTGGA	96
Neoehrlichia mikurensis		Neo_mik_groEL_R	TTCCGGTGTACCATAAGGCTT	
		Neo_mik_groEL_P	AGATGCTGTTGGATGTACTGCTGGACC	
Coxiella burnetii	*idc*	Co_bu_icd_F	AGGCCCGTCCGTTATTTTACG	74
		Co_bu_icd_R	CGGAAAATCACCATATTCACCTT	
		Co_bu_icd_P	TTCAGGCGTTTTGACCGGGCTTGGC	

	IS*1111*	Co_bu_IS111_F	TGGAGGAGCGAACCATTGGT	86
		Co_bu_IS111_R	CATACGGTTTGACGTGCTGC	
		Co_bu_IS111_P	ATCGGACGTTTATGGGGATGGGTATCC	
Ehrlichia canis	*dsb*	Eh_ca_dsb_F	AATACTTGGTGAGTCTTCACTCA	110
		Eh_ca_dsb_R	GTTGCTTGTAATGTAGTGCTGC	
		Eh_ca_dsb_P	AAGTTGCCCAAGCAGCACTAGCTGTAC	
Ehrlichia spp.	16S rRNA	Neo_mik_16S_R	GCAACGCGAAAAACCTTACCA	98
		Neo_mik_16S_R	AGCCATGCAGCACCTGTGT	
		Neo_mik_16S_P	AAGGTCCAGCCAAACTGACTCTTCCG	
Francisella tularensis	*tul4*	Fr_tu_tul4_F	ACCCACAAGGAAGTGTAAGATTA	76
		Fr_tu_tul4_R	GTAATTGGGAAGCTTGTATCATG	
		Fr_tu_tul4_P	AATGGCAGGCTCCAGAAGGTTCTAAGT	
	fopA	Fr_tu_fopA_F	GGCAAATCTAGCAGGTCAAGC	91
		Fr_tu_fopA_R	CAACACTTGCTTGAACATTTCTAG	
		Fr_tu_fopA_P	AACAGGTGCTTGGGATGTGGGTGGTG	
Hepatozoon spp.	18S rRNA	Hepa_spp_18S_F	ATTGGCTTACCGTGGCAGTG	175
		Hepa_spp_18S_R	AAAGCATTTTAACTGCCTTGTATTG	
		Hepa_spp_18S_S	ACGGTTAACGGGGGATTAGGGTTCGAT	
Rickettsia conorii	23S-5S ITS	Ri_co_ITS_F	CTCACAAAGTTATCAGGTTAAATAG	118
		Ri_co_ITS_R	CGATACTCAGCAAAATAATTCTCG	
		Ri_co_ITS_P	CTGGATATCGTGGCAGGGCTACAGTAT	
Rickettsia slovaca	23S-5S ITS	Ri_slo_ITS_F	GTATCTACTCACAAAGTTATCAGG	138
		Ri_slo_ITS_R	CTTAACTTTTACTACAATACTCAGC	
		Ri_slo_ITS_P	TAATTTTCGCTGGATATCGTGGCAGGG	
Rickettsia massiliae	23S-5S ITS	Ri_ma_ITS_F	GTTATTGCATCACTAATGTTATACTG	128
		Ri_ma_ITS_R	GTTAATGTTGTTGCACGACTCAA	
		Ri_ma_ITS_P	TAGCCCCGCCACGATATCTAGCAAAAA	
Rickettsia helvetica	23S-5S ITS	Ri_he_ITS_F	AGAACCGTAGCGTACACTTAG	79
		Ri_he_ITS_R	GAAAACCCTACTTCTAGGGGT	
		Ri_he_ITS_P	TACGTGAGGATTTGAGTACCGGATCGA	
Rickettsia felis	orfB	Ri_fel_orfB_F	ACCCTTTTCGTAACGCTTTGC	163
		Ri_fel_orfB_R	TATACTTAATGCTGGGCTAAACC	
		Ri_fel_orfB_P	AGGGAAACCTGGACTCCATATTCAAAAGAG	
Rickettsia aeschlimannii	ITS	Rick_aesch_ITS_F	CTCACAAAGTTATCAGGTTAAATAG	134

		Rick_aesch_ITS_R	CTTAACTTTTACTACGATACTTAGCA	
		Rick_aesch_ITS_P	TAATTTTTGCTGGATATCGTGGCGGGG	
Rickettsia spp.	gltA	Rick_spp_gltA_F	GTCGCAAATGTTCACGGTACTT	78
		Rick_spp_gltA_R	TCTTCGTGCATTTCTTTCCATTG	
		Rick_spp_gltA_P	TGCAATAGCAAGAACCGTAGGCTGGAT	
Theileria spp.	18S rRNA	Th_an_18S_F	GCGGTAATTCCAGCTCCAATA	126
		Th_an_18S_R	AAACTCCGTCCGAAAAAAGCC	
		Th_an_18S_P	ACATGCACAGACCCCAGAGGGACAC	
Escherichia coli	*eae*	eae-F2	CATTGATCAGGATTTTTCTGGTGATA	102
		eae-R	CTCATGCGGAAATAGCCGTTA	
		eae-P	ATAGTCTCGCCAGTATTCGCCACCAATACC	
Rhipicephalus sanguineus sensu lato	ITS-2	Rhi_san_ITS2_F	TTGAACGCTACGGCAAAGCG	110
		Rhi_san_ITS2_R	CCATCACCTCGGTGCAGTC	
		Rhi_san_ITS2_P	ACAAGGGCCGCTCGAAAGGCGAGA	
Tick spp.	16S rRNA	Tick_spp_16S_F	AAATACTCTAGGGATAACAGCGT	99
		Tick_spp_16S_R	TCTTCATCAAACAAGTATCCTAATC	
		Tick_spp_16S_P	CAACATCGAGGTCGCAAACCATTTTGTCTA	

Table S3

Pairwise comparison of 16S nucleotide sequences (aligned over 429 bp) of ticks determined herein. Nucleotide similarity and percentage differences are given above and below the diagonal, respectively.

Tick species	Sequence ID (GenBank accession no.)	191	187	290	355	192	30	116	302	357	298	7	225
Haemaphysalis punctata	191 (MT799944)	-	0.884	0.882	0.884	0.887	0.818	0.829	0.829	0.831	0.801	0.801	0.806
Hs. sulcata	187 (MT799946)	11.6	-	0.985	0.988	0.99	0.818	0.828	0.828	0.83	0.819	0.822	0.826
Hs. sulcata	290 (MT799948)	11.8	1.5	-	0.992	0.995	0.811	0.823	0.823	0.826	0.814	0.81	0.814
Hs. sulcata	355 (MT799949)	11.6	1.2	0.8	-	0.997	0.813	0.826	0.826	0.828	0.817	0.812	0.817
Hs. sulcata	192 (MT799947)	11.3	1	0.5	0.3	-	0.816	0.828	0.828	0.83	0.819	0.814	0.819
Hyalomma anatolicum	30 (MT799950)	18.2	18.2	18.9	18.7	18.4	-	0.861	0.861	0.863	0.847	0.849	0.852
Rhipicephalus microplus	116 (MT799951)	17.1	17.2	17.7	17.4	17.2	13.9	-	0.995	0.997	0.901	0.884	0.887
Rh. microplus	302 (MT799952)	17.1	17.2	17.7	17.4	17.2	13.9	0.5	-	0.997	0.898	0.882	0.884
Rh. microplus	357 (MT799953)	16.9	17	17.4	17.2	17	13.7	0.3	0.3	-	0.901	0.884	0.887
Rh. haemaphysaloides	298 (MT799956)	19.9	18.1	18.6	18.3	18.1	15.3	9.9	10.2	9.9	-	0.931	0.933
Rh. turanicus	7 (MT799954)	19.9	17.8	19	18.8	18.6	15.1	11.6	11.8	11.6	6.9	-	0.992
Rh. turanicus	225 (MT799955)	19.4	17.4	18.6	18.3	18.1	14.8	11.3	11.6	11.3	6.7	0.8	-

Table S4

Pairwise comparison of *cox1* nucleotide sequences (aligned over 673 bp) of ticks determined herein. Nucleotide similarity and percentage differences are given above and below the diagonal, respectively.

Tick species	Sequence ID (GenBank accession no.)	30	116	302	7	225	18	298	230	340	191	187	192	355
Hyalomma anatolicum	30 (MT80311)	-	0.823	0.821	0.815	0.826	0.82	0.82	0.824	0.826	0.802	0.808	0.806	0.811
Rhipicephalus microplus	116 (MT800322)	17.7		0.998	0.861	0.87	0.864	0.86	0.855	0.857	0.817	0.818	0.817	0.82
Rh. microplus	302 (MT800323)	17.9	0.2	-	0.86	0.869	0.863	0.858	0.854	0.855	0.818	0.82	0.818	0.821
Rh. turanicus	7 (MT800312)	18.5	13.9	14	-	0.982	0.986	0.893	0.878	0.879	0.829	0.823	0.818	0.818
Rh. turanicus	225 (MT800313)	17.4	13	13.1	1.8	-	0.992	0.895	0.887	0.888	0.841	0.83	0.829	0.826
Rh. turanicus	18 (MT800314)	18	13.6	13.7	1.4	0.8	-	0.888	0.879	0.881	0.835	0.824	0.823	0.82
Rh. haemaphysaloides	298 (MT800315)	18	14	14.2	10.7	10.5	11.2	-	0.924	0.925	0.827	0.833	0.833	0.832
Rh. haemaphysaloides	230 (MT800316)	17.6	14.5	14.6	12.2	11.3	12.1	7.6	-	0.998	0.835	0.827	0.827	0.829
Rh. haemaphysaloides	340 (MT800317)	17.4	14.3	14.5	12.1	11.2	11.9	7.5	0.2	-	0.836	0.829	0.829	0.83
Haemaphysalis punctata	191 (MT800318)	19.8	18.3	18.2	17.1	15.9	16.5	17.3	16.5	16.4	-	0.851	0.848	0.848
Hs. sulcata	187 (MT800319)	19.2	18.2	18	17.7	17	17.6	16.7	17.3	17.1	14.9	-	0.989	0.988
Hs. sulcata	192 (MT800320)	19.4	18.3	18.2	18.2	17.1	17.7	16.7	17.3	17.1	15.2	1.1	-	0.992
Hs. sulcata	355 (MT800321)	18.9	18	17.9	18.2	17.4	18	16.8	17.1	17	15.2	1.2	0.8	-

Table S5

Pairwise comparison of ITS-2 nucleotide sequences (aligned over 273 bp) of ticks belonging to *Rhipicephalus* and *Hyalomma* genera determined herein. Nucleotide similarity and percentage differences are given above and below the diagonal, respectively.

**Tick species**	**Sequence ID (GenBank accession no.)**	**7**	**298**	**302**	**30**
Rhipicephalus turanicus	7 (MT818226)	-	0.972	0.913	0.734
Rh. haemaphysaloides	298 (MT818227)	2.8	-	9.8	0.752
Rh. microplus	302 (MT818223)	8.7	0.902	-	0.72
Hyalomma anatolicum	30 (MT818222)	26.6	24.8	28	-
